# Molecular Mechanism for Cellular Response to β-Escin and Its Therapeutic Implications

**DOI:** 10.1371/journal.pone.0164365

**Published:** 2016-10-11

**Authors:** Dominik Domanski, Oliwia Zegrocka-Stendel, Anna Perzanowska, Malgorzata Dutkiewicz, Magdalena Kowalewska, Iwona Grabowska, Dorota Maciejko, Anna Fogtman, Michal Dadlez, Katarzyna Koziak

**Affiliations:** 1 Laboratory of Mass Spectrometry, Institute of Biochemistry and Biophysics, Polish Academy of Sciences, Warsaw, Poland; 2 Centre for Preclinical Research and Technology, Department of Immunology, Biochemistry and Nutrition, Medical University of Warsaw, Warsaw, Poland; 3 Department of Molecular and Translational Oncology, Maria Sklodowska-Curie Memorial Cancer Center and Institute of Oncology, Warsaw, Poland; 4 Department of Cytology, Faculty of Biology, University of Warsaw, Warsaw, Miecznikowa 1, 02–096 Warsaw, Poland; 5 Laboratory for Microarray Analysis CORELAB, Institute of Biochemistry and Biophysics, Polish Academy of Sciences, Warsaw, Poland; University of Sheffield, UNITED KINGDOM

## Abstract

β-escin is a mixture of triterpene saponins isolated from the horse chestnut seeds (*Aesculus hippocastanum* L.). The anti-edematous, anti-inflammatory and venotonic properties of β-escin have been the most extensively clinically investigated effects of this plant-based drug and randomized controlled trials have proved the efficacy of β-escin for the treatment of chronic venous insufficiency. However, despite the clinical recognition of the drug its pharmacological mechanism of action still remains largely elusive. To determine the cellular and molecular basis for the therapeutic effectiveness of β-escin we performed discovery and targeted proteomic analyses and *in vitro* evaluation of cellular and molecular responses in human endothelial cells under inflammatory conditions. Our results demonstrate that in endothelial cells β-escin potently induces cholesterol synthesis which is rapidly followed with marked fall in actin cytoskeleton integrity. The concomitant changes in cell functioning result in a significantly diminished responses to TNF-α stimulation. These include reduced migration, alleviated endothelial monolayer permeability, and inhibition of NFκB signal transduction leading to down-expression of TNF-α—induced effector proteins. Moreover, the study provides evidence for novel therapeutic potential of β-escin beyond the current vascular indications.

## Introduction

β-escin is a mixture of triterpene saponins isolated from horse chestnut seeds (*Aesculus hippocastanum*, L.). Although ethnopharmacological research provides evidence for its broad use to treat numerous diverse disorders, including bladder diseases, cough, diarrhea, dysmenorrhea and tinnitus [1], its current use is restricted mainly to venotonic and venoprotective indications. anti-inflammatory and anti-edematous properties Indeed, randomized controlled trials confirmed the effectiveness of β-escin for the treatment of chronic venous insufficiency (CVI) [2, 3].

The clinical reports on vascular efficacy of β-escin draw particular attention to improved microcirculation, reduced vascular permeability, increased venous tone and venous return, all which lead to edema reduction (reviewed in [4]). It has been suggested that the observed effects result from protection of endothelial cells from hypoxia and inflammatory stimuli provided by the drug [5]. In fact, as shown in preclinical studies, β-escin conserves ATP during oxygen shortage [6], decreases histamine response [5] and cytokine release [7], attenuates serotonin-induced capillary hyperpermeability [8], suppresses extravasation and leukocyte migration [9] and preserves endothelial cell morphology [7]. Worth mentioning are also data indicating antioxidant potential of β-escin [10, 11] and its inhibitory effect on hyaluronidase, an enzyme implicated in the pathogenesis of CVI [12]. In more recent studies inflammation attenuating properties of β-escin has been linked to its modulatory effect on the TNF-α-mediated inflammatory pathways [13].

Despite the therapeutic significance of β-escin and the popularity of the drug which in the United States and Europe remains one of the best-selling herbal extracts accounting for 226 million U.S. dollar-market in 2014 (IMS Kilochem), its exact mechanism of action remains unknown [14]. In the present study we applied a broad experimental approach, including global discovery-type and targeted proteomic methods in conjunction with cellular biology tools to identify novel pathways underlying the protective effects of β-escin in human endothelial cells under inflammatory conditions. The obtained results indicate that the vascular anti-inflammatory mechanism of β-escin involves disturbances in cholesterol homeostasis leading to cytoskeletal perturbations followed by decreased NFκB activation.

## Methods

### Cell culture

Human Umbilical Vein Endothelial Cells (HUVEC, sex unknown, Lonza) were cultured in the EBM-2 (Lonza) supplemented with endothelial growth supplement mix (EGM-2 SingleQuot Kit Supplements and Growth Factors, Lonza) under standard cell culture condition (37°C, 5% CO_2_). Cells were harvested using Accutase (PAA Laboratories). All described experiments were performed with cells of passage four from at least three donors. Unless stated otherwise, the cells were treated for 24 h with DMSO-solubilized β-escin (Nobilus Ent) with or without stimulation of recombinant human TNF-α (rhTNF-α, 10 ng/ml, R&D Systems) for the last 6 h of the experiment. As DMSO concentration in cell culture media did not exceed 0.015%, i.e. its effect of HUVEC was negligible [15], DMSO controls were not included in the experimental protocols.

### Cell viability and proliferation assays

To reliably evaluate viability and proliferative response of cells we used two complementary methods. DNA synthesis in replicating cells was assessed with the BrdU Cell Proliferation Assay (Calbiochem, QIA58), while the ability of intracellular esterases to convert calcein AM, a nonfluorescent substrate into calcein, a fluorescent product was used to evaluate the cell number (Ultrasensitive Cell Proliferation Assay, Calbiochem, QIA128). Briefly, 7 x10^3^ cells/100 μl/well seeded into a 96-well culture dish were allowed to attach overnight prior to the treatment. BrdU was incubated with cells for 24 h, which was followed by a fixative/denaturing step, 1 h anti-BrdU antibody (1∶100) and subsequent 30 min horseradish peroxidase-conjugated goat anti-mouse staining. Incorporated BrdU was detected spectrophotometrically at dual wavelengths of 450–540 nm. Accumulation of fluorescent calcein was determined 1.5 h after supplementing cell culture with calcein AM using a fluorescent plate reader with 485 nm excitation and 520 nm emission filters. In both assays the total incubation time with β-escin was 48 h.

### Cell migration assay

The rate of cell migration was measured with the BD BioCoat™ Angiogenesis System: Endothelial Cell Migration (BD Biosciences, cat. no 354144) strictly according to the manufacturer’s instructions. Briefly, confluent HUVEC were serum starved for 4 h and seeded on migration inserts (1x10^5^ cells/250 μμl). After 22-hour treatment with β-escin with or without 6-hour TNF-α stimulation, the cells on inserts were stained with Calcein AM Fluorescent Dye solution (BD Biosciences) and fluorescence of migrated cells was read directly in a fluorescence plate reader with 485 nm excitation and 520 nm emission filters (Perkin Elmer).

### Vascular permeability assay

The In Vitro Vascular Permeability Assay Kit (Chemicon) was used to determine HUVEC monolayer permeability. All steps of the procedure were performed according to the manufacturer’s instructions. Briefly, 1.8 x 10^5^ cells were plated on the insert, cultured until confluency, and treated with β-escin for 24 h. TNF- α was used as permeability factor for the last 6 h of the cell culture. To evaluate the permeability treatment fluorescein isothiocyanate-conjugated dextran was added to the upper chambers for 20 minutes, then the medium from the lower chambers was harvested and its florescence intensity measured with 485 nm excitation and 535 nm emission filters.

### Cytoskeletal staining

For cytoskeletal staining, HUVEC were washed in PBS, fixed with 3% paraformaldehyde and permeabilized with 0.05% Triton X-100. Polymerized, filamentous actin (F-actin) was visualized by staining with tetramethylrhodamine B isothiocyanate (TRITC)-phalloidin (Sigma-Aldrich). For actin and tubulin co-staining, HUVEC were washed, fixed and permeabilized, as described above. Following incubation with TRITC-phalloidin, primary monoclonal anti-α-tubulin antibody (Sigma-Aldrich) and fluorochrome-conjugated secondary antibody (Alexa Fluor 488) were used. DNA dye DRAQ5 was used to localize the nucleus. Coverslips were mounted using fluorescence mounting medium (DakoCytomation). Samples were analyzed using confocal microscopy (Axiovert 100M, Zeiss) with the LSM 510 META application.

### Evaluation of globullar/fibrillar actin ratio

Determination of actin monomers (globular actin, G-actin) and actin filaments (polymerized, filamentous actin, F-actin) ratio was carried out using the G-actin/F-actin In Vivo Assay kit (Cytoskeleton) strictly according to the manufacturer’s protocol. Briefly, 1x10^6^ cells were lysed, homogenized and centrifuged at 400 g for 5 minutes. The obtained supernatant containing total cellular actin was centrifuged at 100,000 x g for 1 h at 37°C to separate F-actin (pellet) from soluble G-actin (supernatant). F-actin pellets were resuspended in ice cold dH_2_O containing 10 μM cytochalasin D to the same volume as the G-actin supernatant. Equal volumes of samples were analyzed by Western blotting with the anti-actin antibody (Sigma Aldrich, cat. no A3854). The ratio of F-actin to G-actin in each sample was calculated following the densitometry analysis performed with the use of ImageJ software (http://imagej.nih.gov).

### Cholesterol quantification

Total cholesterol concentration was measured using the fluorometric Amplex Red Cholesterol assay (Invitrogen). Briefly, 6 x 10^6^ cells were lysed in 400 μl PBS by three consecutive freeze/thaw cycles at -80°C and centrifuged at 14,000 x g for 10 minutes in 4°C. Reactions containing 150 μM Amplex® Red reagent, 1 U/ml HRP, 1 U/ml cholesterol oxidase, 0,1 μM cholesterol esterase and 50 μl of cell lysates or known amount of cholesterol for standard curve, were incubated at 37°C for 30 minutes. Fluorescence was measured with a fluorescence microplate reader (excitation: 560 nm; emission: 590 nm). Each assay run included no-cholesterol negative control and 10 μM H_2_O_2_ positive control, as per manufacturer’s instructions. Obtained values were normalized to total cellular protein concentration measured by the Lowry method (Biorad).

### Transcription factors quantification

To detect and quantify NFκB p50 and p65 transcription factors activation we used NFκB p50 and NFκB p65 ELISA Kits with Nuclear Extraction Kits (Affymetrix) following the manufacturer’s instructions. For each experimental sample, nuclear extracts were obtained from 6 x10^6^ cells grown to confluence. Protein concentration was measured by the Lowry method (Biorad).

### Global proteomic analysis and multiple reaction monitoring (MRM) analysis of targeted proteins

Details of the materials and methods are presented in the Supporting Information (S1 Methods). Briefly, the global discovery-type proteomic analysis was carried out using the Isobaric Tags for Relative and Absolute Quantitation (iTRAQ) method which enables the identification and relative quantitation of all detectable proteins present in the samples.[16] Proteins were identified with Mascot and differentially expressed proteins were assessed using the Diffprot and MScan software tools with estimation of statistical significance [17]. A minimum number of two peptides with a false-discovery rate <1% were used for confident identification of a protein in the iTRAQ experiment. Subsequently, 32 proteins were selected to be subjected to MRM analysis for accurate targeted verification of iTRAQ results and further analysis of larger samples sets. Each protein was represented by one to three unique peptides. For 23 selected proteins, stable isotope-labeled standard (SIS) peptides were used for increased accuracy and stable-isotope-dilution-MRM (SID-MRM) was performed, and nine proteins of the cholesterol biosynthesis pathway were assessed by MRM without the use of SIS peptides (MRM) but with normalization to beta and gamma actin peptides.

### Statistics

All data are presented as mean ± SEM. When error bars do not appear in the figures, errors are within the size of the symbol used there. All statistical analyses were carried out with GraphPad Prism 5.04 software. Data were analyzed by one-way analyses of variance (ANOVA) followed by the post hoc Tukey multiple range test, unless indicated otherwise. Differences between groups were rated significant at a probability error (p) of < 0.05. For MRM results t- test with Benjamini -Hochberg correction was used to asses significant differences between treatment groups (p -values <0.05).

### Pathway analysis

For canonical pathways and network analyses proteins with significant alterations in quantity were submitted to Ingenuity Pathway Analysis (Ingenuity Systems, Redwood City, CA; http://www.ingenuity.com).

## Results

### Effect of β-escin on endothelial cell viability and proliferation

**Fig 1:**
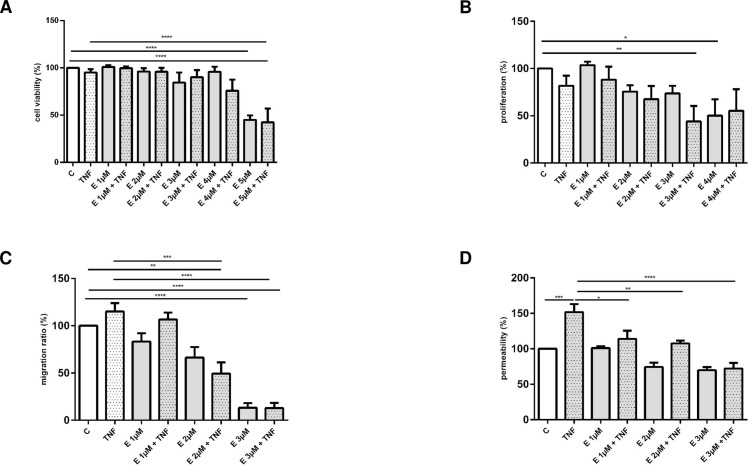
**The effects of β-escin on quiescent and TNF-α-treated vascular endothelial cells: viability (Panel A), proliferation (Panel B), migration (Panel C) and permeability (Panel D) of HUVEC presented as a percentage of fluorescence (Panels A, C and D) or absorbance readouts (Panel B) in the control, untreated cells.** Abbreviations: C -untreated cells; TNF—TNF-α-treated cells; E—β-escin-treated cells. Results are expressed as mean ±SEM of three experiments with cells obtained from at least three donors. Data were analyzed by one-way analyses of variance (ANOVA) followed by the post hoc Tukey multiple range test. *p < 0.05; **p < 0.01; ***p < 0.001

Structurally β-escin belongs to saponins which are known for their membrane-permeabilising properties [18]. Their lytic action on erythrocyte membranes is one of the best known features shared by all molecules of this chemical class. In order to evaluate the effect of β-escin on HUVEC viability and proliferation we measured the number of viable cells and the BrdU incorporation rate. As shown in Fig 1A, β-escin induced toxicity assessed following 48 h treatment was observed in concentrations above 4 μM and the effect was not significantly influenced with TNF-α. β-escin influence on HUVEC proliferation rate was quantified after 48 h of culture, became statistically significant at the 3 μM for cells treated with β-escin and TNF-α and at the 4 μμM concentration for cells cultured with β-escin without TNF-α (Fig 1B).

### Effect of β-escin on endothelial cell migration

The ability of a cell to migrate is essential in numerous vascular responses, including vascular inflammation and angiogenesis. β-escin induced decline in migration of both quiescent and TNF-α stimulated cells reached statistical significance at the 2 μM concentration (Fig 1C).

### Effect of β-escin on endothelial layer integrity

The integrity of the endothelial cell monolayer is one of the key conditions necessary to protect vascular homeostasis. As demonstrated in Fig 1D, β-escin protected the endothelial layer against TNF-α-induced permeability. The observed effect was statistically significant already at the 1 μM concentration of β-escin.

### Cholesterol content

**Fig 2:**
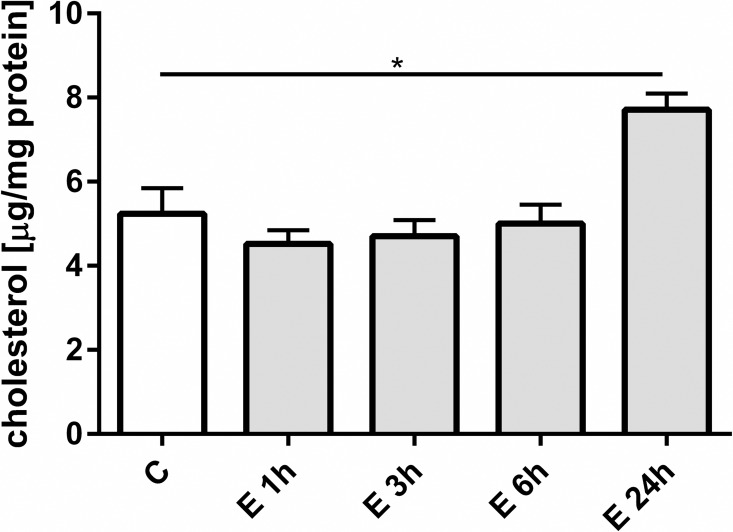
The change of total intracellular intracellular cholesterol content in HUVEC following treatment with 3 μM β-escin. Abbreviations: C—untreated HUVEC; E—β-escin treated cells. The results are expressed as the mean ±SEM of three independent experiments with cells obtained from at least six donors. Data were analyzed by one-way analyses of variance (ANOVA) followed by the post hoc Tukey multiple range test. *p < 0.05

β-escin-dependent loss of membrane cholesterol has been previously solidly confirmed [19]. Results presented in Fig 2 present a statistically significant rise in total cellular cholesterol content following 24 h of β-escin treatment.

### β-escin disrupts the actin cytoskeleton

Well-established evidence links the integrity of the actin cytoskeleton with normal cellular responses. Moreover, it has been documented that the organization of cell actin is cholesterol-sensitive. Disturbed cholesterol homeostasis and reduced migratory response of β-escin treated endothelial cells prompted us to investigate its effect on the actin cytoskeleton in HUVEC.

**Fig 3:**
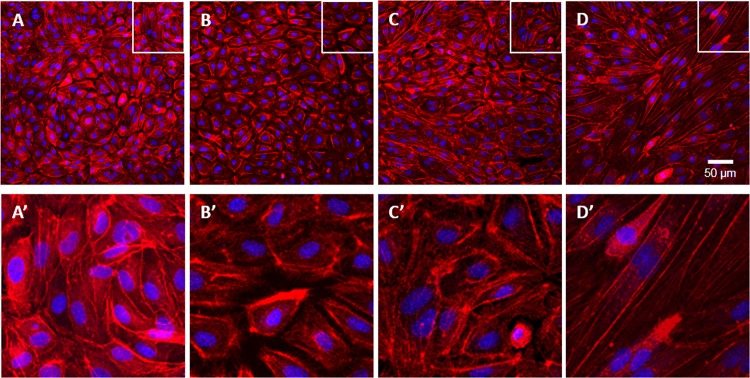
HUVEC actin cytoskeleton remodeling following treatment with increasing concentrations of β-escin. Upper panel: actin staining of: A—control cells; B, C, D—β-escin-treated cells (1, 2 and 3 μM, respectively). Lower panel (A’, B’, C’, D’): magnification of selected fragments. Similar results were observed in three independent experiments with cells obtained from three donors.

**Fig 4:**
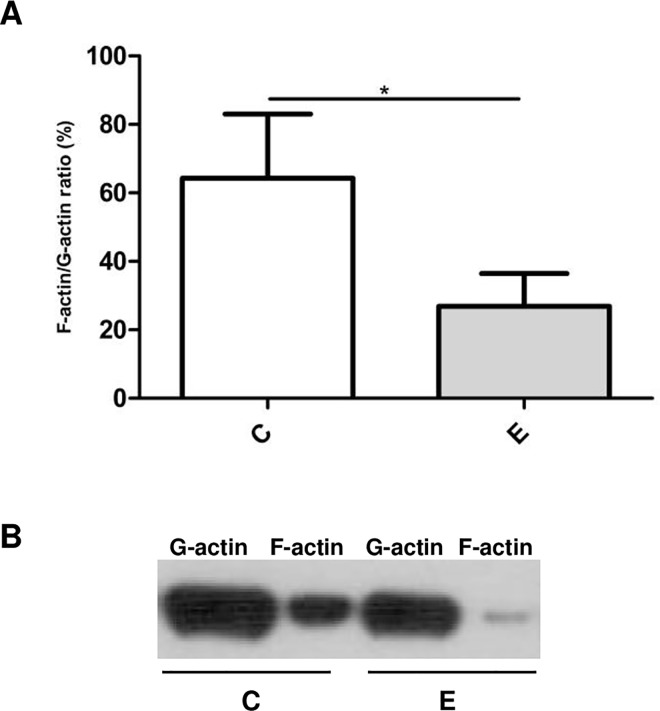
Quantitation of the globular / fibrillar actin ratio (G-actin / F-actin) in HUVEC following incubation with 3 μM β-escin. A. Data shown were obtained in three independent experiments performed with cells from five donors. The paired t-test (GraphPad Prism v.6.01) was used for statistical analysis. *p < 0.05. B. Typical assay results showing changes in the amount of G-actin and F-actin in HUVEC treated with 3 μM β-escin. Abbreviations: C—untreated HUVEC; E—β-escin treated cells.

As demonstrated with two different techniques, β-escin induced depolymerization of actin filaments. Staining of fibrillar actin with phalloidin revealed morphological changes and actin cytoskeleton remodeling, as demonstrated by confocal microscopy (Fig 3). In addition, the assessment of fibrillar and globular actin fractions demonstrated an increase in the G-actin / F-actin ratio from 1.8 in control to 4.2 in β-escin treated samples (Fig 4). Neither TNF-α nor β-escin, alone or in the combined treatment, had a statistically significant effect on the total cell actin content as shown by MRM analysis of both beta and gamma actin (data not shown). No effect of β-escin on the total actin content suggests that the modifications of actin cytoskeleton are not related to altered actin synthesis.

### Differential proteome analysis of the β-escin-treated endothelial cells using iTRAQ

Results of the aforementioned functional analyses of endothelial cells cultured in the presence of β-escin prompted us to perform a discovery-type proteomic study using the iTRAQ technique. We applied this method to discover proteins involved in β-escin induced cellular responses and to elucidate the anti-inflammatory mechanisms of β-escin action in more detail.

Although at a low concentration (1 μM) β-escin did not induce significant changes in the protein profile (data not shown), at the 3 μM concentration it strongly affected the protein expression pattern (S1 Fig and Table A in S1 Methods). Importantly, we also revealed 75 differentially expressed proteins in β-escin and TNF-α treated cells as compared to TNF-α stimulated cells (S1 Fig and Table B in S1 Methods).

**Fig 5:**
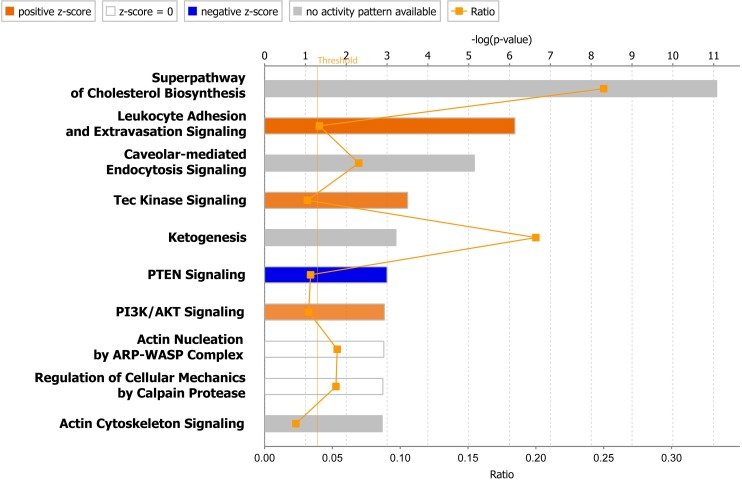
Top ten canonical pathways associated with β-escin action on HUVEC activated with TNF-α. The orange line represents the threshold p-value (0.05). For pathways analysis proteins with significant alterations in quantity were submitted to Ingenuity Pathway Analysis. The taller are the bars the more significant is the probability of association of proteins from the dataset with the canonical pathway.

To assemble and evaluate protein–protein associations and to identify functional protein linkages we next analyzed the differentially expressed proteins using IPA. The ten canonical pathways most significantly modulated with β-escin treatment, presented in Fig 5, shed more light on the cellular and biochemical data obtained in the study. The findings emphasize the importance of the increased cholesterol synthesis in β-escin-treated endothelial cells, as presented in more detail in S2 Fig.

### Verification of iTRAQ results using the MRM technique

**Fig 6:**
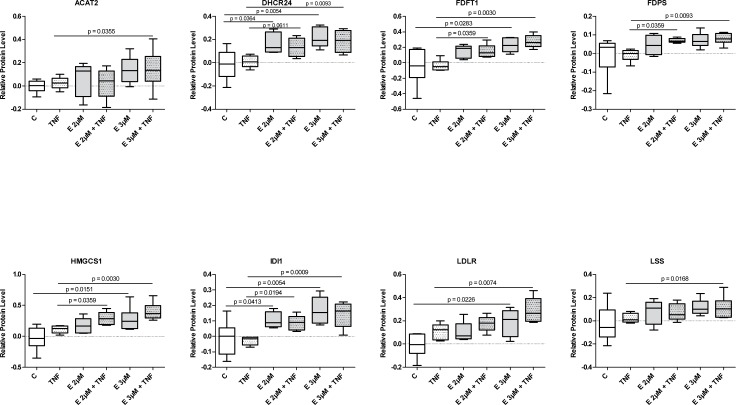
MRM analysis of proteins involved in the cholesterol biosynthesis pathway and transport. Relative protein amounts normalized to control (with median and range) are derived from the geometric mean of signals for all peptides of a given protein. Significant differences between treatment groups with a p -values <0.05, as determined using t- test with Benjamini -Hochberg correction are indicated.

To verify the iTRAQ results we carried out MRM targeted MS analysis of selected proteins. The advantage of MRM analysis includes higher specificity and detection accuracy combined with the ability to process larger sample sets. The increased sample throughput allowed us to analyze the effect of β-escin at 2 and 3 μM concentrations, with and without TNF-α, on HUVEC obtained from six donors. The MRM analysis was composed of 32 proteins selected from the iTRAQ-indicated pool of molecules with altered expression following β-escin and/or TNF-α treatment (Table 1, Fig 6 and S3 Fig).

**Table 1:** SID-MRM analysis of TNF-α and/or β-escin effect in HUVEC. For SID-MRM tests proteins were selected on the basis of iTRAQ indications. Cells obtained from six different donors were used. Significant alternations according to Benjamini-Hochberg corrected p-values determined with t-test on respective regression coefficients are indicated by asterisks (***—p value of 0.0001 to 0.001; **—0.001 to 0.01 and *—0.01 to 0.05; ns–non significant).

iTRAQ indication	Protein name	Change upon TNF-α treatment	Change upon β-escin treatment	Change upon β-escin treatment of TNF-α induced cells
affected by TNF-α	Intercellular adhesion molecule 1 (ICAM-1 / ICAM1)	↑ ***	**ns**	**ns**
	Plasminogen activator inhibitor 1 (PAI-1 / SERPINE1)	↑ ***	**ns**	**ns**
	von Willebrand factor (vWF / VFW)	↓ ***	**ns**	**ns**
	Superoxide dismutase [Mn], mitochondrial (SOD2)	↑ ***	**ns**	↓ *
	Low affinity cationic amino acid transporter 2 (CAT-2 / SLC7A2)	↑ ***	**ns**	↓ *
	Tumor necrosis factor alpha-induced protein 2 (TNFAIP2)	↑ ***	**ns**	↓ **
	DnaJ homolog subfamily A member 1 (DNAJA1)	↑ **	**ns**	↓ **
	EH domain-containing protein 1 (EHD1)	↑ *	**ns**	↓ *
	COUP transcription factor 1 & 2 (COUP-TF1, COUP-TF2 / NR2F1, NR2F2)	↓ **	↓ *	**ns**
	Thrombospondin-1 (THBS1)	**ns**	**ns**	**ns**
affected by TNF-α and β-escin	Vascular cell adhesion protein 1 (VCAM-1 / VCAM1)	↑ ***	**ns**	↓ **
	E-selectin (SELE)	↑ ***	**ns**	**ns**
	Endothelial protein C receptor (EPCR / PROCR)	**ns**	↓ *	↓ **
	Multimerin-1 (MMRN1)	**ns**	**ns**	↓ *
	Hedgehog-interacting protein (HHIP)	**ns**	**ns**	**ns**
affected by β-escin	Cathepsin B (CTSB)	**ns**	↓ **	↓ ***
	Cathepsin Z (CTSZ)	**ns**	↓ **	↓ ***
	Palmitoyl-protein thioesterase 1 (PPT-1 / PPT1)	**ns**	↓ *	↓ ***
	CD63 antigen (LAMP-3 / CD63)	**ns**	↑ **	↑ ***
	Amyloid beta A4 protein (APP)	**ns**	↑ *	↑ **
	Integral membrane protein 2B (ITM2B)	**ns**	↑ **	↑ **
	Matrix metalloproteinase-14 (MMP-14 / MMP14)	**ns**	↑ ***	↑ **
	Podocalyxin (PODXL)	**ns**	↑ *	↑ **

The MRM protein panel consisted of a selection of: 1) proteins that have been long recognized as hallmarks of TNF-α activation of endothelial cells and are classically associated with systemic inflammation such as SELE, ICAM1 and VCAM1 and potential novel endothelial TNF-α-responsive proteins indicated by our global analysis, *i*.*a*. DNAJA1 and HHIP, 2) proteins of the cholesterol biosynthesis and metabolism pathway and 3) proteins unaffected by TNF-α but responding to β-escin.

#### 1) β-escin influence on TNF-α responsive proteins

SID-MRM analysis confirmed changes in abundance of 11 out of 15 TNF-α responsive proteins indicated by the iTRAQ analysis (Table 1 and S3 Fig). Importantly, SID-MRM analysis proved quantitative TNF-α dependent changes in the cellular content of three known TNF-α responders, TNFAIP2, CAT-2 and COUP-TF1/COUP-TF2, for which p-values in the iTRAQ analysis did not reach statistical significance, *i*.*e*. the p-values were above 0.05 despite fold changes >1.3. Therefore, the results of MRM analysis indicated that the iTRAQ-Diffprot cut-off value of p-value set in our experiments at <0.05 was rather conservative, and validated our additional listing of proteins in Tables A and B in S1 Methods with changes above 1.3-fold supported by four or more peptides and with a Diffprot p-value >0.05 and <0.67. Moreover, SID-MRM analysis revealed β-escin influence on six TNF-α-responsive proteins, which was not previously indicated by the global iTRAQ analysis (S3 Fig), *i*.*e*. TNFAIP2, SOD2, CAT-2, DNAJA1, COUP-TF1/COUP-TF2 and EHD1.

#### 2) Cholesterol biosynthesis pathway

We performed MRM analysis also to validate the biochemical data and results of the iTRAQ analysis which revealed a previously unknown, potent up-regulation of the cholesterol biosynthesis pathway evoked with β-escin treatment (Fig 6). The protein levels of seven biosynthetic enzymes (acetyl-CoA acetyltransferase/ ACAT2, hydroxymethylglutaryl-CoA synthase/HMGCS1, isopentenyl-diphosphate delta-isomerase 1/IDI1, squalene synthase/FDFT1, delta(24)-sterol reductase/DHCR24, farnesyl pyrophosphate synthase/FDPS and lanosterol synthase/LSS) and two molecules involved in cholesterol transport (low-density lipoprotein receptor/LDLR and vigilin/HDLBP) were evaluated. The results confirmed a clear stimulatory effect of β-escin on all the analyzed cholesterol biosynthesis enzymes and LDLR in TNF-α stimulated cells. Although less pronounced, the stimulatory effect of β-escin on enzymes involved in cholesterol biosynthesis and transport was detected also in quiescent cells treated with the drug. The results support the iTRAQ data, except for the transport-involved protein HDLBP, previously suggested to be weakly decreased by β-escin-TNF-α treatment, now shown to be unaffected by any treatments.

#### 3) β-escin effect on non-TNF- α responding proteins

We performed SID-MRM analysis also to verify changes in the profile of eight proteins not involved in cholesterol pathway which appeared in the iTRAQ data to be β-escin and non-TNF-α responding (S3 Fig). The obtained results confirmed a β-escin-evoked increase in the amount of APP, ITM2B, CD63, podocalyxin and MMP-14 and a reduction in the abundance of cathepsin B, cathepsin Z and PPT-1 in β-escin treated cells. For the latter group, TNF-α had a further reducing effect which was not observed before in the iTRAQ analysis.

### β-escin dependent NFκB dysfunction

**Fig 7:**
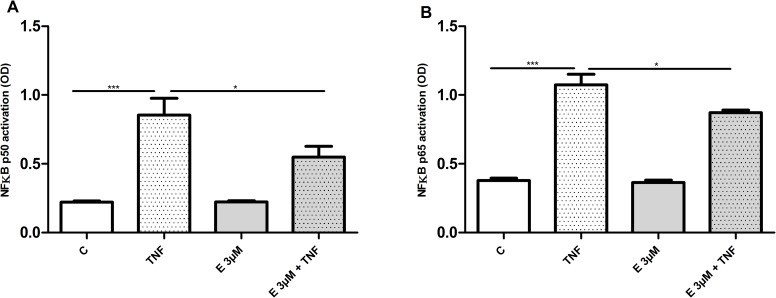
Effect of β-escin on NFκB p50 and p65 activation. Abbreviations: C—untreated cells; TNF—TNF-α-treated cells; E– 3 μM-escin-treated cells. The results are expressed as the mean ±SEM of four independent experiments with HUVEC obtained from four donors. Data were analyzed by one-way analyses of variance (ANOVA) followed by the post hoc Tukey multiple range test. *p < 0.05; ***p < 0.001

β-escin has been recently recognized as an inflammation attenuating agent, modulating the TNF-α-mediated inflammatory pathways [13]. To investigate transcriptional events initiated by HUVEC exposure to β-escin we measured the activity of NFκB, the master transcriptional regulator of the inflammatory pathway. As shown in Fig 7, a decrease in TNFα-induced NFκB activation, illustrated by inhibition of nuclear translocation of p50 and p65, was detected in cells treated with 3 μM β-escin.

## Discussion

This study provides a thorough insight into the molecular and cellular mechanisms of the vascular anti-inflammatory effect of β-escin. We show that this plant-based drug significantly affects endothelial cell structure and function by disrupting the actin cytoskeleton and decreasing expression of TNF-α—induced effector proteins. Our results suggest that the observed effects may be linked to the β-escin induced disturbances of cholesterol homeostasis.

The ability of β-escin to interfere with cell membranes has been recognized as one of its most characteristic features. In fact, a large number of biological effects of β-escin and other saponins, has been ascribed to pore formation, release of cellular compounds and cell lysis. The mechanism leading to perturbation of membrane integrity is not yet fully understood, but several reports indicate the saponin-dependent loss of membrane cholesterol as a key event leading to cytotoxicity contributing to hemolytic activity of natural extracts with high saponin content [19].

The current study verifies many of the commonly acknowledged β-escin toxicity concerns. Although saponin induced cell damage and hemolysis have been considered as an important obstacle in their clinical applications, we demonstrate that the pharmacological properties of β-escin are not related to reduced endothelial cell viability. The thorough evaluation of β-escin effect on HUVEC facilitated the proper estimation of non-toxic concentrations that appeared to be significantly lower than in many of the previous studies [20].

Recently published data confirms that β-escin belongs to a group of potent membrane toxic saponins inducing significant membrane cholesterol release already after one hour of treatment [19]. However, to the best of our knowledge, an augmented abundance of proteins involved in cholesterol biosynthesis and increased cellular cholesterol content demonstrated in this study have so far remained an unrecognized cellular effects of this drug. It could be speculated that this phenomenon represents a strong compensatory response to β-escin-induced efflux of cellular cholesterol, but a direct effect of the drug on cholesterol biosynthesis cannot be excluded. Interestingly, an enhanced cholesterol biosynthesis following saponin treatment has been previously reported for one of the ginseng saponins, ginsenoside-Rb_1_ and a structure-function relationship significant for exerting this effect has been proposed [21].

A large body of evidence points towards actin as one of the important links connecting cholesterol to cytoskeleton organization and cellular function [22–24]. Therefore, β-escin induced disturbances in cholesterol homeostasis could explain perturbations of actin cytoskeleton integrity followed by rapid changes in cell functioning revealed in our analyses. As our proteomic analysis indicated no influence of β-escin on actin synthesis, improper actin assembly observed in β-escin treated cells could result from reduced expression of several well-studied actin-associated proteins, including β-catenin, transgelin and tropomyosins TPM1 and TPM2. An integral and functional actin cytoskeleton is recognized as a critical element in inflammatory responses. Numerous studies showed that actin cytoskeleton disruption down-regulated inflammatory events, including expression, production and secretion of inflammatory mediators, without affecting cell viability [25–27]. In endothelial cells exposed to pro-inflammatory agonists, such as TNF-α or extracellular nucleotides, properly assembled actin cytoskeleton is prerequisite for *e*.*g*. cell spreading, adhesion, migration and invasion [28–30]. It is also well documented that actin plays a central role in regulating the width of the intercellular clefts in endothelial cells, thereby controlling the paracellular pathway of vascular permeability [31]. Therefore, a defective actin organization in β-escin treated cells revealed in our study could be considered as an important phenomenon underlying anti-inflammatory properties of this drug. Interestingly, while down-regulating most of the analyzed proteins known to affect cellular functions through their association with actin cytoskeleton, β-escin increases the content of podocalyxin.Functionally, this cell surface sialomucin acts as an anti-adhesin playing an important role in cell migration, adhesion and lumen formation [32, 33]. However, recently published data demonstrated a previously unrecognized key role of podocalyxin in the interactions between endothelial cells and matrix that regulate vascular integrity [34]. Thus, β-escin—mediated protection of endothelial layer integrity under inflammatory conditions demonstrated in our study could be at least partially related to podocalyxin accumulation.

A β-escin—dependent decrease in the amount of multiple lysosomal molecules involved in lipid metabolism, of which cathepsins constitute the most abundant group, appears as yet another important element in the vascular anti-inflammatory β-escin action. The precise mechanism by which β-escin could influence lysosomal fate is not known, but it has been shown that both a decrease and an increase in cholesterol content may modulate lysosomal stability [35, 36]. The role of cathepsins in lipid degradation, and particularly in cholesterol efflux, their involvement in inflammation, contribution to neovascularization and cardiovascular diseases has been very well established [37, 38].

The perturbation of cholesterol content has also a significant impact on signal transduction. The results of this study identify several signal transduction processes as relevant to β-escin action, *i*.*e*. Tec kinase, PTEN, PI3K/Akt and actin cytoskeleton signaling. However, it is caveolar mediated endocytosis signaling that has been classified as one of the canonical pathways most significantly affected with β-escin treatment. This observation remains in concordance with the established data recognizing signaling defects and an inhibition of endocytosis resulting from a disruption of caveolae as the major cholesterol-sensitive processes [39–41]. More recently, another plausible mechanism of cholesterol dependent signaling dysregulation was suggested [42]. As cholesterol inserted between fatty acid acyl chains of biological membranes modulates their fluidity [43], it might affect the 3-D conformation and function of transmembrane proteins. A significant reduction in the nuclear translocation of NF-κB subunits in β-escin treated cells stimulated with TNF-α presented in this study validates the data published previously for significantly higher and, as shown in this study, β-escin concentrations toxic for endothelial cells [44]. A blockade of NF-κB activation suggests a defect in TNF-α signaling which could be linked either to inefficient TNF-α receptor function or to an disturbed NF-κB translocation process. Both of the TNF-α receptors present in endothelial cells, TNFR1 and TNFR2 contain only a single membrane-spanning domain and thus the conformational disturbances due to disturbed membrane fluidity seem less probable. However, as the rates of actin polymerization and depolymerization are critical determinants of NF-κB nuclear import and, as a consequence, of transcriptional activity of NF-κB dependent genes [45], it is more probable that the observed reduction in nuclear transport of NF-κB subunits results from disturbances in actin arrangement rather than from TNF-α receptor dysfunction.

The results of proteomic analyses further strengthen the data on the detected inhibition of the NF-κB pathway. A decreased content of TNF-α-induced effector proteins involved in different stages of endothelial cell activation, such as VCAM-1, TNFAIP2, CAT-2, SOD2, EHD1 and DNAJA1, detected in β-escin treated cells strongly suggests that β-escin acts as an inhibitor of the TNF-α-regulated processes at the initial activation stage of the NF-κB pathway (see S4 Fig).

Interestingly, β-escin treatment has a profound influence also on proteins that are not directly associated with any of the above mentioned pathways. Of particular importance seems an increase in the content of two proteins playing a major role in the pathogenesis of Alzheimer’s disease: APP, a direct precursor of β-amyloid, and ITM2B (BRI2), which is a physiological suppressor of β-amyloid production [46]. This seemingly surprising result remains in concordance with a constantly growing evidence reinforcing and extending the mechanistic insight into cholesterol homeostasis and amyloidogenesis [47–51]. The identification of a β-escin effect on amyloidogenesis could be considered particularly beneficial in the context of a TNF-α related increase in amyloidogenic APP processing [52]. Depiction of the mechanism underlying β-escin influence on APP processing and the potential role of the drug in prevention and/or therapy of Alzheimer's disease merits more thorough studies.

The precise recognition of the role in β-escin exerted anti-inflammatory effects and other cellular functions played by all the 75 β-escin-responsive proteins identified in the proteomic analyses requires extended studies. Certain disturbances in the protein profile may represent compensatory responses to disturbances in cholesterol content and actin depolimerization, as discussed above and as it has been suggested for *e*.*g*. MMP14 (MT1-MMP) [53]. However, we cannot exclude other underlying mechanisms. Functional links between β-escin treatment and the change in cellular content of other important for endothelial function, such as CD63, needs to be established.

In summary, the global analysis of β-escin effects on endothelial cells suggests that the β-escin induced perturbation in cholesterol homeostasis may be considered as the triggering event in a cascade of cellular responses leading to cytoskeletal disarrangements followed by a decreased NFκB activation and down-expression of TNF-α—induced effector proteins. defining mechanisms of pharmacological effectiveness of the drug. Our results indicate that the vascular anti-inflammatory mechanism of β-escin involves disturbances in cholesterol homeostasis leading to cytoskeletal perturbations followed by decreased NFκB activation. Moreover, our data provide evidence for novel therapeutic potential of β-escin beyond current vascular indications.

## Supporting Information

S1 Fig Venn diagram showing the number of proteins exhibiting significant abundance alterations as a result of the experimental treatments. Proteins with a q -value of <0.05, and proteins with a fold change ≥ 1.3, identified with ≥ 4 peptides and having a q value <0.67. Abbreviations: Control–untreated cells; TNF–TNF-α-treated cells; E–cells treated with 3 μM β-escin.
(TIF)

S2 Fig The stimulatory effect of β-escin on cholesterol biosynthesis pathway observed in TNF-α treated cells. The induced enzymes are marked in red.
(PDF)

S3 Fig MRM analysis of proteins expressed differentially in TNF-α activated HUVEC treated with 3 μM β-escin. Relative protein amounts normalized to control (with median and range) are derived from the geometric mean of signals for all peptides of a given protein as indicated in Table B in S1 Methods. Significant differences between treatment groups with a p -values <0.05, as determined using t- test with Benjamini -Hochberg correction are indicated.
(TIF)

S4 Fig A summary of the biological processes, pathways and proteins affected by β -escin action. (Panel A) Arrow-ended lines indicate activation of a process or protein, blunt-ended lines indicate inhibition and square-ended lines indicate both or an interaction. Up and down arrows next to the proteins show significant changes in quantity observed as a result of TNF -α (black arrows) and 3 μM β-escin (red arrows) in the iTRAQ and/or MRM analyses. Proteins measured in our study are indicated in black with those in gray added for clarification of a pathway. Full protein names are indicated in the text. (Panel B) Graphical abstract of the main study results.
(TIF)

S1 Methods (DOCX)
