# Supplementary figures and images for: Molecular Mechanism for Cellular Response to β-Escin and Its Therapeutic Implications
Source: PLoS One. 2016 Oct 11;11(10):e0164365. doi: 10.1371/journal.pone.0164365 (PMC5058498; doi:10.1371/journal.pone.0164365)

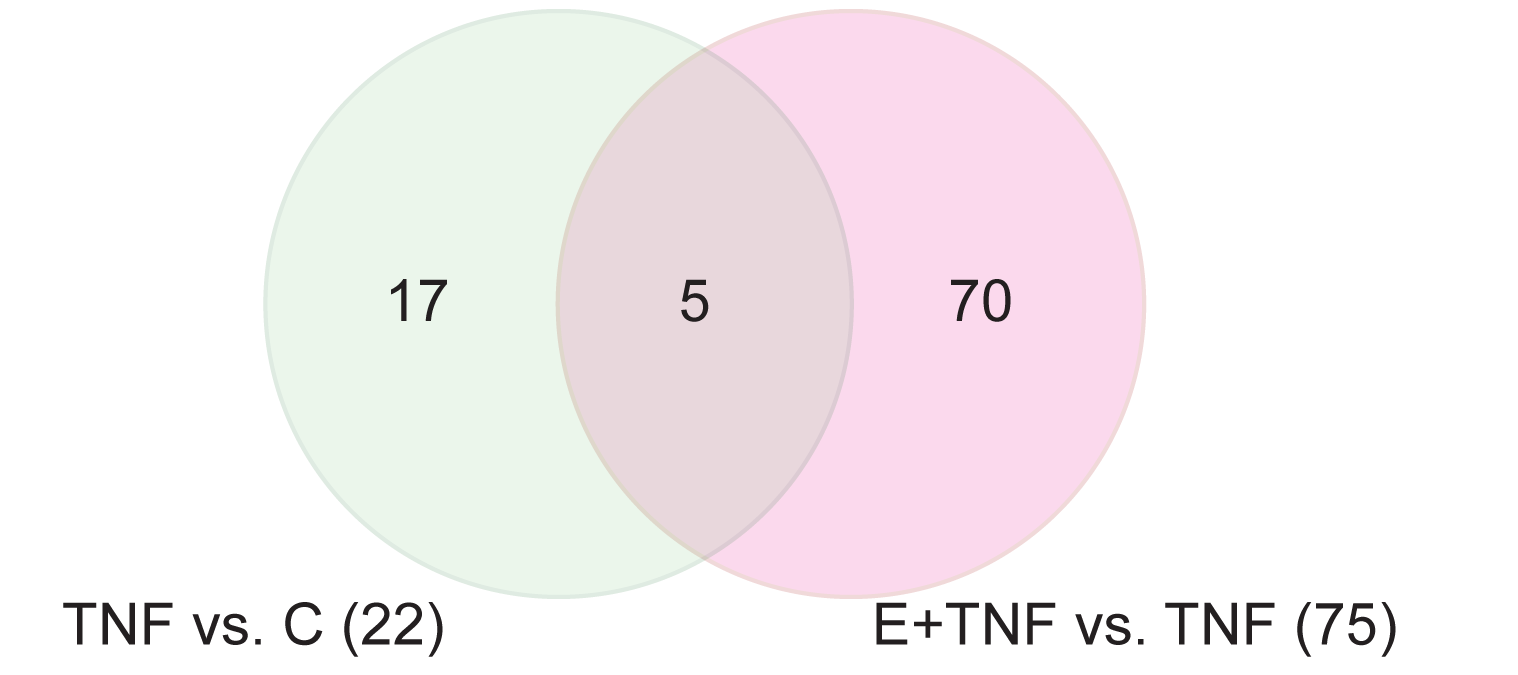

Supplement: S1 Fig — Proteins with a q -value of <0.05, and proteins with a fold change ≥ 1.3, identified with ≥ 4 peptides and having a q value <0.67. Abbreviations: Control–untreated cells; TNF–TNF-α-treated cells; E–cells treated with 3 μM β-escin. (TIF) [file pone.0164365.s001.tif]

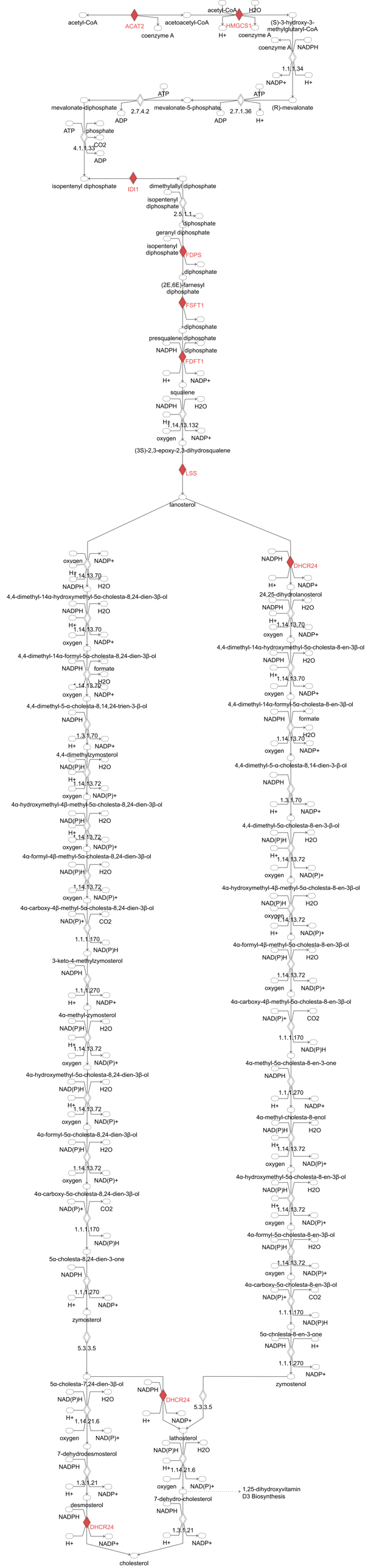

Supplement: S2 Fig — The induced enzymes are marked in red. (PDF) [file pone.0164365.s002.pdf]

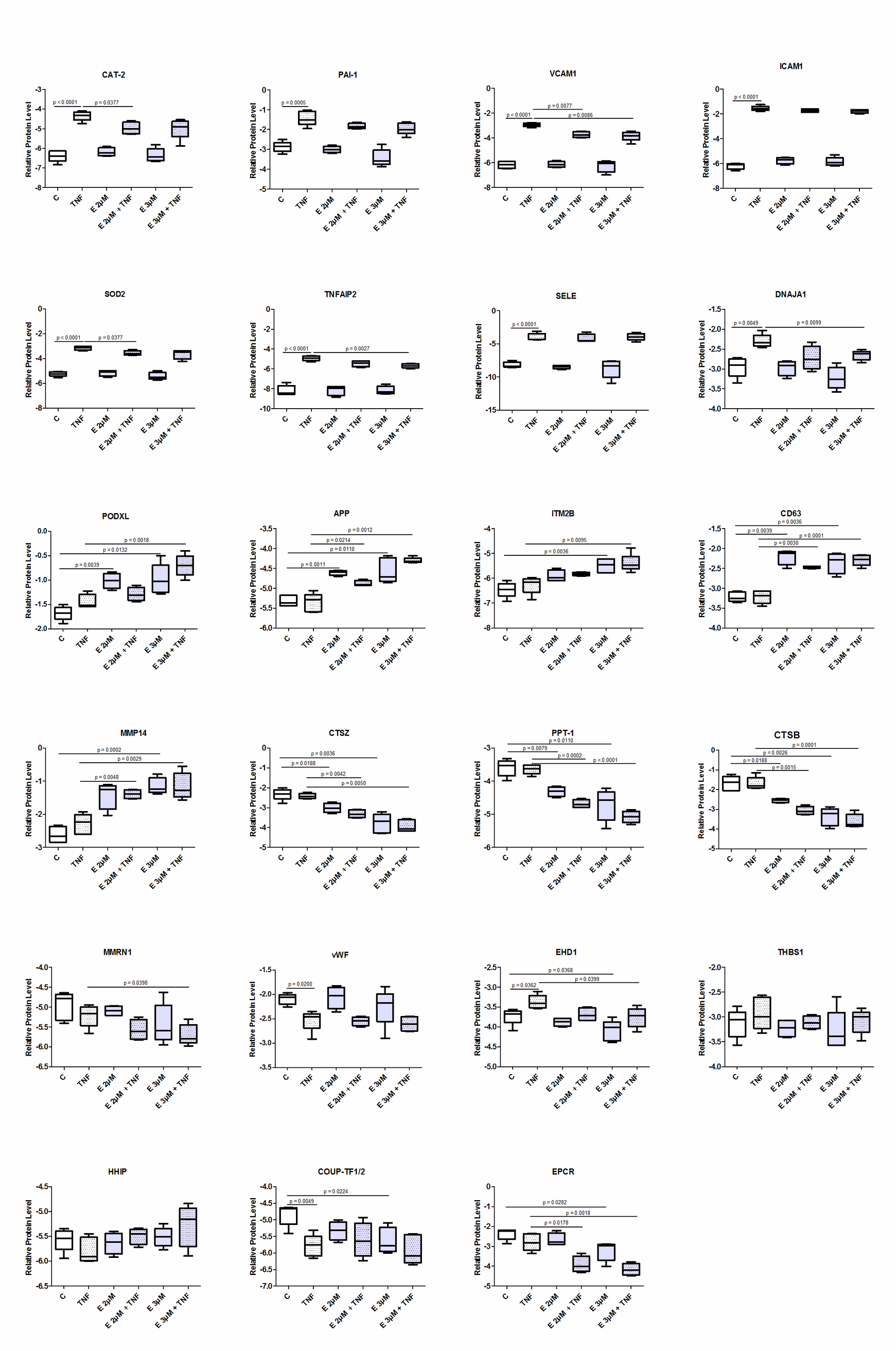

Supplement: S3 Fig — Relative protein amounts normalized to control (with median and range) are derived from the geometric mean of signals for all peptides of a given protein as indicated in Table B in S1 Methods. Significant differences between treatment groups with a p -values <0.05, as determined using t- test with Benjamini -Hochberg correction are indicated. (TIF) [file pone.0164365.s003.tif]

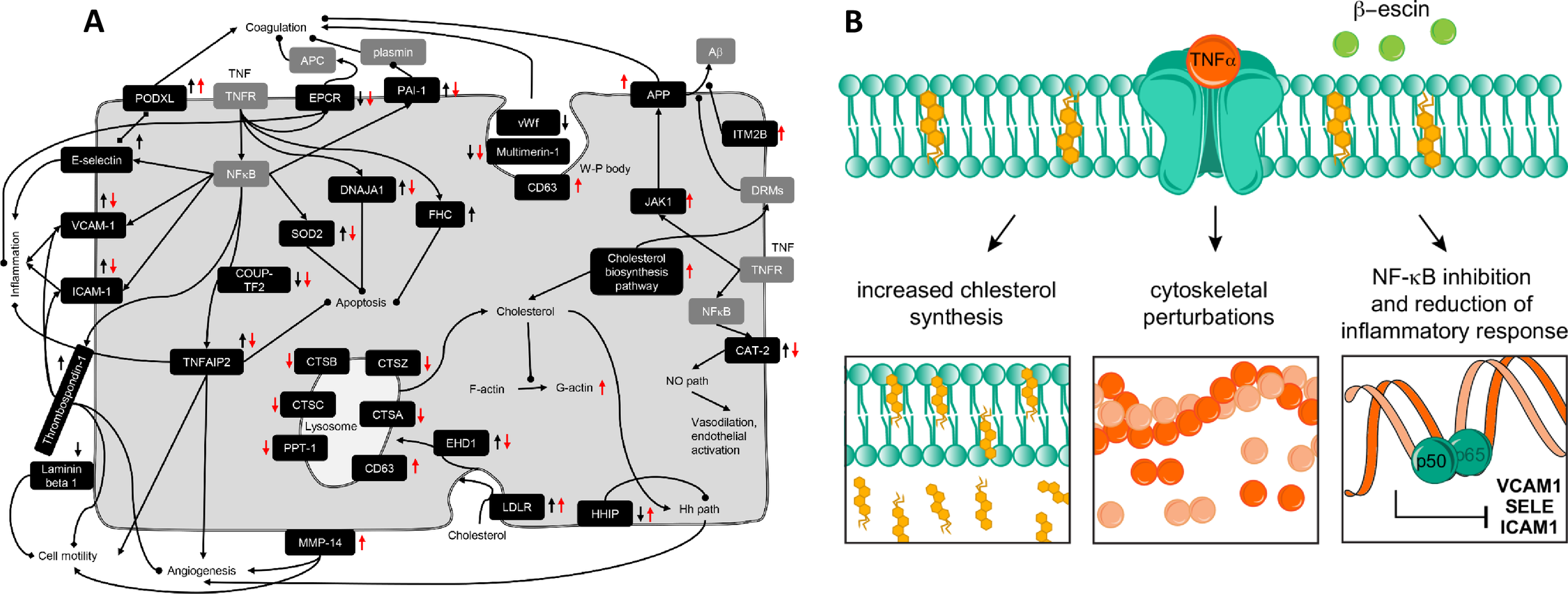

Supplement: S4 Fig — (Panel A) Arrow-ended lines indicate activation of a process or protein, blunt-ended lines indicate inhibition and square-ended lines indicate both or an interaction. Up and down arrows next to the proteins show significant changes in quantity observed as a result of TNF -α (black arrows) and 3 μM β-escin (red arrows) in the iTRAQ and/or MRM analyses. Proteins measured in our study are indicated in black with those in gray added for clarification of a pathway. Full protein names are indicated in the text. (Panel B) Graphical abstract of the main study results. (TIF) [file pone.0164365.s004.tif]
