# Supplementary material for: Molecular Mechanism for Cellular Response to β-Escin and Its Therapeutic Implications
Source: PLoS One. 2016 Oct 11;11(10):e0164365. doi: 10.1371/journal.pone.0164365 (PMC5058498; doi:10.1371/journal.pone.0164365)
Supplement: S1 Methods — (DOCX) [file pone.0164365.s005.docx]

**S1 Methods**

**Sample preparation for Isobaric Tags for Relative and Absolute Quantitation (iTRAQ) analysis**

HUVEC pellets (~1^e^6 cells) were obtained by centrifugation of harvested cells at 300 x g for 6 minutes at 24°C and freezing at -80°C. Frozen cell pellets were lysed by re-suspension in 300 µl cold 1% sodium deoxycholate (NaDOC) in 25 mM ammonium bicarbonate (AmmBic, Sigma-Aldrich) and boiling for 5 minutes. Nucleic acids were then digested with Benzonase nuclease (75 U, Sigma-Aldrich) for 30 minutes at 4°C. Lysates were centrifuged at 12,000 x g for 10 minutes at 4°C and supernatant was stored at -80°C. Protein quantification was performed using the bicinchoninic acid (BCA, Thermo Fisher Scientific, Rockford) protein assay according to the manufacturer’s instructions.

**Isobaric Tags for Relative and Absolute Quantitation (iTRAQ) analysis**

Three treatment groups of HUVEC were analyzed using iTRAQ: control cells (C), cells treated only with TNF-α (10 ng/ml) for 6 hours (TNF) and cells treated with β-escin (3 µM) for 24 hours with stimulation with TNF-α (10 ng/ml) for the last 6 hours (E+TNF). Three independent biological replicate sets were analyzed with three separate iTRAQ analyses. Each biological replicate was composed of a different donor cell pool composed of three unrelated HUVEC donors. Each treatment sample was then analyzed with two technical replicates within the 8-plex iTRAQ assay (AB SCIEX). Protein extracts (100 µg) were precipitated with 1 ml cold acetone overnight at -20°C and pelleted by centrifugation at 6000 x g for 10 min. Protein digestion and iTRAQ labeling was done as per manufacturer’s instructions using the 8-plex iTRAQ assay and iTRAQ Reagent and Buffer Kits (AB SCIEX). In brief, protein pellets were solubilized in 0.5 M triethylammonium bicarbonate with 0.1% sodium dodecyl sulphate (SDS) and sonication for 15 minutes. Samples were then reduced with *tris*(2-carboxyethyl)phosphine (TCEP) and cysteines blocked with methyl methanethiosulfonate (MMTS). Proteins were digested with sequencing grade trypsin for 16 hours at 37°C at a 1:12.5 enzyme:protein ratio (Promega). Subsequently, samples were labeled with iTRAQ tags as follows: 113 and 114 for C, 115 and 116 for TNF, and 117 and 118 for E+TNF. Labeled peptides were pooled and their volume was reduced using a SpeedVac (CentriVap). Samples were diluted with 0.1% formic acid (FA, Sigma-Aldrich) to 1 ml and desalted using solid phase extraction (SPE) on an Oasis HLB 1cc (10 mg) cartridge (Waters, Milford, MA, USA) as follows. The resin was rinsed with methanol (J.T.Baker) and equilibrated with water (LC-MS grade, J.T. Baker). Samples were loaded, washed with 1.0 ml of water and eluted in 300 µl 75% acetonitrile (ACN, LC-MS grade, J.T.Baker), 0.1% FA. Fractions were evaporated to dryness using a SpeedVac and re-suspended in 170 µl of 8 M urea, 50 µM dithiotreitol (DTT), 0.002% bromophenol blue in 50 mM Tris/HCl pH 8 (Sigma-Aldrich) with 2 µl of 3-11 NL ampholytes (IPG buffer, GE Healthcare). The mixture was added to a polyacrylamide gel strip with an immobilized nonlinear pH gradient 3-11 NL (18cm, GE Healthcare) and rehydrated overnight at room temperature in an IPG BOX (GE, Healthcare). The isoelectric focusing separation (IEF) was carried out for 200 000Vh using an IPGphor3 Ettan (GE, Healthcare). The strip was cut into 18 1 cm parts and each was extracted with 100 µl of 0.1% trifluoroacetic acid (TFA, Sigma-Aldrich), 2% ACN for 1 hour. Extracted peptides were stored at -80°C till LC-MS/MS analysis.

**LC-MS/MS analysis**

A Waters nanoACQUITY UPLC system was used for peptide separation prior to MS/MS analysis. Mobile phase A consisted of 0.1% FA and mobile phase B was ACN/0.1% FA. Twenty µl of the post IEF peptide extract was injected onto a reversed-phase trapping column (180 µm x 20 mm, C18, 5 µm, ACQUITY UPLC Symmetry, Waters) using mobile phase A. Peptides were transferred to a reversed-phase analytical column (75 µm x 250 mm, nanoACQUITY UPLC BEH130 C18 Column, 1.7 µm, Waters) and separated using a 250 nl/min flow rate and a gradient from 3-33% mobile phase B over 150 minutes. The column was directly coupled to the ion source of an LTQ Orbitrap Velos (Thermo Fisher Scientific) which worked in the regime of data-dependent MS to MS/MS switch, with the following criteria: HCD fragmentation, one MS scan followed by a maximum of 5 MS/MS scans, 1.5kV capillary voltage and data acquired in positive polarity mode.

**Qualitative MS/MS data processing and iTRAQ quantitative analysis**

Data processing was performed as previously described [[1](#_ENREF_1)]. Briefly, Mascot Distiller (version 2.3.2.0, Matrix Science, London, UK) was used to pre-process MS/MS data with default value settings for iTRAQ labeling. This included the merging of redundant spectra and removal of noisy spectra. Next, a two-step database search procedure [[2](#_ENREF_2)] was carried out. A first, less restrictive database search of the data was conducted using the MASCOT search engine and the Swiss-Prot *Homo sapiens* database (from 03.2012) which enables the calculation of MS and MS/MS measurement errors and recalibration of the data for a repeated stringent MASCOT search. The initial search parameters were set as follows: enzyme, trypsin; fixed modification, cysteine modification by MMTS as well as iTRAQ labeling of the N-terminus of peptides and of lysine side chains; variable modifications - oxidation (M); max missed cleavages – 1, peptide tolerance of 100 ppm, MS/MS tolerance of 0.2 Da. The data was calibrated and filtered using the MScan program [[2](#_ENREF_2)] with a queries threshold of 10. The merged file of all the IEF fractions was then again searched using MASCOT against the Swiss-Prot database supplemented with a decoy database of randomized sequences mixed in, enabling the calculation of the false discovery rate (FDR). This procedure provided *q*-value estimates for each peptide spectrum match (PSM) in the dataset. All PSMs with *q*-values > 0.01 were removed from further analysis. Additional search parameters to those mentioned above now included a peptide tolerance of 6 ppm and fragment ion tolerance of 0.2 Da. Further data filtration was performed using MScan with the following criteria: at least two peptide observations per protein, proteins that matched the same set of peptides were clustered into one protein group, and proteins identified by a subset of peptides from another protein were removed from the analysis. MS/MS spectra of peptides meeting the above acceptance criteria were subjected to the quantitative analysis step to obtain a list of differentially expressed proteins as indicated by the iTRAQ reporter tags. The differentially expressed proteins list with estimation of statistical significance of a single protein ratio was acquired using the in-house program Diffprot [[3](#_ENREF_3)] as described previously [[1](#_ENREF_1)].

**Selection of peptides for multiple reaction monitoring**

The selection of peptides for multiple reaction monitoring (MRM) was performed according to the criteria described previously [[4](#_ENREF_4)]. Briefly, tryptic peptides best suited for MRM analysis were selected using databases such as: UniProt, PeptideAtlas, SwissProt-Expasy, NCBI BLASTp, and the NCBI SNP database with the following criteria: unique peptide sequence to the targeted protein, highly observed in spectral databases, peptide length does not exceed 20 amino acids, avoided easily chemically modifiable residues and sequences prone to modifications, peptides with missed cleavages and low efficiency of digestion were excluded, peptides containing high frequency single-nucleotide polymorphisms were excluded, and peptides containing reported post-translational modifications or with known biological features affecting their accurate measurement were excluded.

For the stable-isotope dilution-MRM (SID-MRM) analysis, the selected peptide sequences were synthesized as stable-isotope labeled standard (SIS) peptides, using isotopically labeled amino acids on the C-terminus: Arg ^13^C_6_; ^15^N_4_ (98% isotopic enrichment) or Lys ^13^C_6_; ^15^N_2_ (98% isotopic enrichment) by JPT Peptide Technologies GmbH as SpikeTides_L using the proprietary SPOT peptide synthesis technology.

**Sample preparation for MRM analysis**

For the MRM analysis HUVEC cells were treated as above, and with β-escin alone without TNF-α stimulation, in addition to the earlier treatments of TNF-α stimulation post β-escin treatment, control cells, and cells treated only with TNF-α. Additionally, β-escin was used at 2 µM and 3 µM concentrations. Each treatment was tested with six HUVEC cultures (biological replicates) originating from six single independent donors, with each cell culture within each treatment being analyzed in two technical replicates. Fifty µg of protein extract was denatured in a final concentration of 1% NaDOC in 100 mM AmmBic. Disulphide bonds were next reduced with 2 µl of 50 mM TCEP (in 100 mM AmmBic) for 1 hour at 60°C and alkylated with 2 µL of 100 mM iodoacetamide (Sigma-Aldrich) for 30 minutes at 37°C in the dark. Sequencing grade trypsin was added to obtain a 1:12.5 enzyme:protein ratio and digestion was carried out for 16 hours at 37°C. To a volume corresponding to 25 µg of digested proteins a mixture of SIS peptides was added to give a an amount of SIS ranging from 50 to 500 fmol/µg of sample for each single SIS peptide and a final 0.33% formic acid concentration to precipitate the NaDOC detergent. NaDOC was removed by centrifugation at 14,000 x g for 10 minutes at room temperature. The supernatant was transferred to a polypropylene HPLC auto-sampler vial and analyzed by nano-LC-MRM.

**Nano-LC-MRM, optimization and analysis**

MRM analysis was performed as described previously [[1](#_ENREF_1)] with minor modifications. In brief, a Waters Xevo TQ mass spectrometer (Waters) coupled to a Waters nanoAcquity UPLC was used with mobile phase A as 0.1% FA in LC-MS grade water (J.T.Baker), and mobile phase B as LC-MS grade ACN with 0.1% FA. Peptides were loaded onto a Waters 2G nanoAcquity UPLC Symmetry C18 trap-column (180 µm x 20 mm, 5 µm) and separated using a 60 min LC run, with a gradient of mobile phase B changing from 1 to 10% from 0 to 10 min. and from 10 to 50% from 10 to 40 min. on a Waters nanoAcquity UPLC BEH130 C18 column (75 µm x 150 mm, 1.7 µm). Other MS instrument parameters included a capillary voltage of 3.5 kV, a purge gas flow of 100 L/h, cone gas flow of 5 l/h, NanoFlow gas set at 2.0 Bar, and a source temperature of 150°C.

The optimization of peptide-specific MRM settings for the SID-MRM analysis was performed as previously described [[1](#_ENREF_1)] to generate the highest signal for each individual peptide and ion fragments. Briefly, using the selected ion recording (SIR) function controlled by the Waters MassLynx V4.1 software the optimal precursor charge and optimal cone voltage were established using injections of pure SIS peptides. Thereafter, optimal collision energy (CE) voltages were determined to obtain the highest possible signals of fragment ions of b- and y-series for both 2+ and 3+ ion charge states within an m/z range of 300 to 1400. For the analysis of cholesterol pathway proteins and actin a standard MRM analysis was performed with default cone voltage and CE settings as suggested for the Waters TQ system and by the Skyline (Ver. 2.5) software, respectively. Five to three optimized transitions that generated the highest signals were selected for each peptide that were free of signal interferences for both the endogenous and heavy (SIS) peptides.

For the SID-MRM analysis the quantity of endogenous peptide is reported as the Peak Area Ratio (PAR) which is the sum of the peak areas of all transitions for the endogenous peptide divided by the sum of the peak areas of transitions of its heavy standard. The addition of equivalent amounts of standard peptides to the analyzed samples and the calculation of PAR enables the normalization of natural peptide relative abundance between samples in terms of MS signal fluctuations and post-digestion sample processing differences. For the MRM analysis of the cholesterol pathway proteins and actin the sum of the total peak areas of five transitions for the endogenous peptide were presented as the relative amounts. In the case of the cholesterol pathway proteins the relative amounts were normalized to the level of actin peptide signals.

LC-MRM-MS analysis was carried out with 1µl of sample injected, corresponding to 1 µg of digested proteins, with blank (0.1% FA) runs in between every sample. All MRM data was processed using the Skyline Ver. 2.5 software with default values for peak integration and Savitzky-Golay peak smoothing. All integrated peaks were manually inspected to ensure correct peak detection and accurate integration. All peptides were targeted using 3 to 5 MRM ion pairs per peptide that were interference free.

**Statistical analysis**

Analysis of SID-MRM data was performed on a set of signals averaged over two technical replicates. For each independent variable - single peptide reading or geometric mean of signals for all peptides of a given protein – a linear predictor has been found with donor, TNF-α treatment and β-escin dose as independent variables. Student's t-test was used to check whether predictor's coefficients were significantly different than zero. Benjamini-Hochberg algorithm was used to adjust p-values for multiple (peptides/proteins) hypothesis testing. The validity of a linear predictor approach was assessed with a Shapiro-Wilk test for residuals. For the purpose of plotting and testing β-escin impact on TNF-α treatment peptides/proteins signal were normalized using donors coefficients. All computations were performed on log2 signal values. Statistical analyses of cholesterol pathway proteins and actin MRM data were performed using VassarStats (Poughkeepsie, NY USA) with p-values estimated using a two-tailed Mann-Whitney U-test. The statistical significance of iTRAQ results was determined using the program Diffprot [[3](#_ENREF_3)].

**References**

1. Bakun M, Niemczyk M, Domanski D, Jazwiec R, Perzanowska A, Niemczyk S, et al. Urine proteome of autosomal dominant polycystic kidney disease patients. Clin Proteomics. 2012;9(1):13. doi: 10.1186/1559-0275-9-13. PubMed PMID: 23228063; PubMed Central PMCID: PMC3607978.

2. Mikula M, Gaj P, Dzwonek K, Rubel T, Karczmarski J, Paziewska A, et al. Comprehensive analysis of the palindromic motif TCTCGCGAGA: a regulatory element of the HNRNPK promoter. DNA Res. 2010;17(4):245-60. doi: 10.1093/dnares/dsq016. PubMed PMID: 20587588; PubMed Central PMCID: PMC2920758.

3. Malinowska A, Kistowski M, Bakun M, Rubel T, Tkaczyk M, Mierzejewska J, et al. Diffprot - software for non-parametric statistical analysis of differential proteomics data. J Proteomics. 2012;75(13):4062-73. doi: 10.1016/j.jprot.2012.05.030. PubMed PMID: 22641154.

4. Mohammed Y, Domanski D, Jackson AM, Smith DS, Deelder AM, Palmblad M, et al. PeptidePicker: a scientific workflow with web interface for selecting appropriate peptides for targeted proteomics experiments. J Proteomics. 2014;106:151-61. doi: 10.1016/j.jprot.2014.04.018. PubMed PMID: 24769191.

**Table A. The effect of β-escin in TNF-α activated HUVEC: differentially expressed proteins identified by iTRAQ analysis**.Three independent biological replicate sets were analyzed with three separate iTRAQ analyses and each biological replicate was composed of cells pooled from three HUVEC donors. Statistical significance for the change is indicated by the *q-value* from the Diffprot software. *Ratio* indicates the direction and magnitude of change, with above 1.0 indicating an increase in protein abundance in the TNF-α-treated cells. *Fold-change* indicates the magnitude of the increase or decrease in abundance. *Peptides* indicates the number of unique peptides for each protein used for identification and the averaged abundance measurement. Appended at the bottom of the list are proteins outside the restrictive statistical cut-off of q-value<0.05, but with a minimum fold-change of 1.3, 4 or more peptides, and q-value <0.67 (see text).

| **Accession number** | **q-value** | **Ratio** | **Fold-change** | **Peptides** | **Protein name** |
| --- | --- | --- | --- | --- | --- |
| **Q9Y287** | 0.0001 | 3.79 | 3.79 | 2 | Integral membrane protein 2B GN=ITM2B |
| **Q09666** | 0.0001 | 0.9 | 1.11 | 204 | Neuroblast differentiation-associated protein AHNAK GN=AHNAK |
| **O00592** | 0.0001 | 1.56 | 1.56 | 6 | Podocalyxin GN=PODXL |
| **Q00341** | 0.0001 | 0.86 | 1.17 | 28 | Vigilin GN=HDLBP |
| **P05556** | 0.0001 | 1.18 | 1.18 | 17 | Integrin beta-1 GN=ITGB1 |
| **Q02952** | 0.0001 | 1.21 | 1.21 | 53 | A-kinase anchor protein 12 GN=AKAP12 |
| **P07858** | 0.0001 | 0.64 | 1.57 | 5 | Cathepsin B GN=CTSB |
| **P19320** | 0.0001 | 0.69 | 1.45 | 23 | Vascular cell adhesion protein 1 GN=VCAM1 |
| **Q96QV1** | 0.0001 | 1.25 | 1.25 | 11 | Hedgehog-interacting protein GN=HHIP |
| **P00491** | 0.0001 | 1.3 | 1.3 | 17 | Purine nucleoside phosphorylase GN=PNP |
| **P05067** | 0.0001 | 2 | 2 | 7 | Amyloid beta A4 protein GN=APP |
| **P21333** | 0.0001 | 0.91 | 1.1 | 115 | Filamin-A GN=FLNA |
| **P16581** | 0.0001 | 1.42 | 1.42 | 14 | E-selectin GN=SELE |
| **P50897** | 0.0001 | 0.62 | 1.62 | 9 | Palmitoyl-protein thioesterase 1 GN=PPT1 |
| **P43121** | 0.0001 | 1.2 | 1.2 | 19 | Cell surface glycoprotein MUC18 GN=MCAM |
| **P78417** | 0.0001 | 1.1 | 1.1 | 16 | Glutathione S-transferase omega-1 GN=GSTO1 |
| **O00469** | 0.0001 | 0.81 | 1.23 | 17 | Procollagen-lysine,2-oxoglutarate 5-dioxygenase 2 GN=PLOD2 |
| **Q9H223** | 0.0003 | 1.11 | 1.11 | 19 | EH domain-containing protein 4 GN=EHD4 |
| **P27105** | 0.0003 | 1.16 | 1.16 | 7 | Erythrocyte band 7 integral membrane protein GN=STOM |
| **P29279** | 0.0004 | 0.73 | 1.36 | 6 | Connective tissue growth factor GN=CTGF |
| **O75923** | 0.0005 | 1.22 | 1.22 | 18 | Dysferlin GN=DYSF |
| **O94832** | 0.0005 | 1.15 | 1.15 | 20 | Myosin-Id GN=MYO1D |
| **P08648** | 0.0005 | 1.18 | 1.18 | 19 | Integrin alpha-5 GN=ITGA5 |
| **Q96HC4** | 0.0005 | 0.89 | 1.13 | 19 | PDZ and LIM domain protein 5 GN=PDLIM5 |
| **Q14392** | 0.0005 | 1.19 | 1.19 | 3 | Leucine-rich repeat-containing protein 32 GN=LRRC32 |
| **P23458** | 0.0005 | 1.32 | 1.32 | 8 | Tyrosine-protein kinase JAK1 GN=JAK1 |
| **Q9UNN8** | 0.0008 | 0.61 | 1.63 | 4 | Endothelial protein C receptor GN=PROCR |
| **Q01995** | 0.001 | 0.75 | 1.33 | 11 | Transgelin GN=TAGLN |
| **Q15392** | 0.0011 | 1.33 | 1.33 | 6 | Delta(24)-sterol reductase GN=DHCR24 |
| **P09493** | 0.0015 | 0.78 | 1.29 | 5 | Tropomyosin alpha-1 chain GN=TPM1 |
| **P05783** | 0.0018 | 0.88 | 1.14 | 12 | Keratin, type I cytoskeletal 18 GN=KRT18 |
| **P17813** | 0.0039 | 1.21 | 1.21 | 15 | Endoglin GN=ENG |
| **Q9NPY3** | 0.0044 | 1.31 | 1.31 | 7 | Complement component C1q receptor GN=CD93 |
| **Q13907** | 0.0045 | 1.3 | 1.3 | 7 | Isopentenyl-diphosphate Delta-isomerase 1 GN=IDI1 |
| **Q9NY15** | 0.0047 | 0.69 | 1.44 | 9 | Stabilin-1 GN=STAB1 |
| **P35241** | 0.0053 | 1.26 | 1.26 | 13 | Radixin GN=RDX |
| **P07951** | 0.006 | 0.69 | 1.45 | 4 | Tropomyosin beta chain GN=TPM2 |
| **P51813** | 0.0064 | 0.85 | 1.18 | 12 | Cytoplasmic tyrosine-protein kinase BMX GN=BMX |
| **P42892** | 0.0064 | 1.16 | 1.16 | 18 | Endothelin-converting enzyme 1 GN=ECE1 |
| **P00352** | 0.0078 | 0.88 | 1.13 | 19 | Retinal dehydrogenase 1 GN=ALDH1A1 |
| **P14324** | 0.0082 | 1.22 | 1.22 | 10 | Farnesyl pyrophosphate synthase GN=FDPS |
| **Q13201** | 0.0096 | 0.83 | 1.21 | 17 | Multimerin-1 GN=MMRN1 |
| **Q92503** | 0.0096 | 1.39 | 1.39 | 5 | SEC14-like protein 1 GN=SEC14L1 |
| **P08729** | 0.0097 | 0.79 | 1.26 | 11 | Keratin, type II cytoskeletal 7 GN=KRT7 |
| **P16284** | 0.0105 | 1.16 | 1.16 | 21 | Platelet endothelial cell adhesion molecule GN =PECAM1 |
| **Q01581** | 0.012 | 1.13 | 1.13 | 12 | Hydroxymethylglutaryl-CoA synthase, cytoplasmic GN=HMGCS1 |
| **P17301** | 0.0123 | 1.1 | 1.1 | 17 | Integrin alpha-2 GN=ITGA2 |
| **Q9BXS4** | 0.0124 | 2.6 | 2.6 | 2 | Transmembrane protein 59 GN=TMEM59 |
| **P14923** | 0.0125 | 1.18 | 1.18 | 12 | Junction plakoglobin GN=JUP |
| **P17302** | 0.0151 | 1.58 | 1.58 | 2 | Gap junction alpha-1 protein GN=GJA1 |
| **O00170** | 0.0153 | 0.81 | 1.24 | 6 | AH receptor-interacting protein GN=AIP |
| **P37268** | 0.0153 | 1.36 | 1.36 | 9 | Squalene synthase GN=FDFT1 |
| **P11717** | 0.0153 | 1.41 | 1.41 | 5 | Cation-independent mannose-6-phosphate receptor GN=IGF2R |
| **P23467** | 0.0165 | 1.32 | 1.32 | 10 | Receptor-type tyrosine-protein phosphatase beta GN=PTPRB |
| **Q05682** | 0.0184 | 0.88 | 1.13 | 18 | Caldesmon GN=CALD1 |
| **Q9UBR2** | 0.0198 | 0.56 | 1.8 | 4 | Cathepsin Z GN=CTSZ |
| **P48449** | 0.0204 | 1.2 | 1.2 | 6 | Lanosterol synthase GN=LSS |
| **Q14254** | 0.0208 | 0.88 | 1.13 | 9 | Flotillin-2 GN=FLOT2 |
| **P49588** | 0.0218 | 0.87 | 1.15 | 27 | Alanine--tRNA ligase, cytoplasmic GN=AARS |
| **P68871** | 0.0229 | 2.1 | 2.1 | 2 | Hemoglobin subunit beta GN=HBB |
| **P08962** | 0.0293 | 1.85 | 1.85 | 2 | CD63 antigen GN=CD63 |
| **P48509** | 0.0323 | 1.34 | 1.34 | 3 | CD151 antigen GN=CD151 |
| **Q9BWD1** | 0.0375 | 1.19 | 1.19 | 8 | Acetyl-CoA acetyltransferase, cytosolic GN=ACAT2 |
| **Q16222** | 0.0377 | 0.82 | 1.22 | 7 | UDP-N-acetylhexosamine pyrophosphorylase GN=UAP1 |
| **Q13310** | 0.042 | 0.9 | 1.11 | 13 | Polyadenylate-binding protein 4 GN=PABPC4 |
| **Q9UDY4** | 0.0422 | 0.9 | 1.12 | 13 | DnaJ homolog subfamily B member 4 GN=DNAJB4 |
| **P10619** | 0.0446 | 0.74 | 1.34 | 5 | Lysosomal protective protein GN=CTSA |
| **Q99613** | 0.0478 | 0.9 | 1.11 | 22 | Eukaryotic translation initiation factor 3 subunit C GN=EIF3C |
| **P50281** | 0.0661 | 1.6 | 1.6 | 4 | Matrix metalloproteinase-14 GN=MMP14 |
| **P55011** | 0.098 | 1.3 | 1.3 | 7 | Solute carrier family 12 member 2 GN=SLC12A2 |
| **P60033** | 0.1738 | 1.6 | 1.6 | 4 | CD81 antigen GN=CD81 |
| **Q9NYL4** | 0.2482 | 0.76 | 1.31 | 4 | Peptidyl-prolyl cis-trans isomerase FKBP11 GN=FKBP11 |
| **P01130** | 0.3237 | 2.12 | 2.12 | 4 | Low-density lipoprotein receptor GN=LDLR |
| **P53634** | 0.5023 | 0.74 | 1.36 | 7 | Dipeptidyl peptidase 1 GN=CTSC |
| **O14964** | 0.5532 | 1.43 | 1.43 | 6 | Hepatocyte growth factor-regulated tyrosine kinase substrate GN=HGS |

**Table B. TNF-α effect on HUVEC: differentially expressed proteins identified by iTRAQ analysis.** Three independent biological replicate sets were analyzed with three separate iTRAQ analyses and each biological replicate was composed of cells pooled from three HUVEC donors. Statistical significance for the change is indicated by the q -value from the Diffprot software. Ratio indicates the direction and magnitude of change, with above 1.0 indicating an increase in protein abundance in the TNF-α -treated cells. Fold-change indicates the magnitude of the increase or decrease in abundance. Peptides indicates the number of unique peptides for each protein used for identification and the averaged abundance measurement. Appended at the bottom of the list are proteins outside the restrictive statistical cut -off of q -value<0.05, but with a minimum fold-change of 1.3, 4 or more peptides, and q-value <0.67 which was observed for the TNF-responsive protein TNFAIP2.

| **Accession number (UniProt)** | **q-value** | **Ratio** | **Fold-change** | **Peptides** | **Protein name** |
| --- | --- | --- | --- | --- | --- |
| **P05121** | 0.0003 | 2.4 | 2.4 | 18 | Plasminogen activator inhibitor 1 GN=SERPINE1 |
| **Q9H4M9** | 0.0003 | 1.23 | 1.23 | 14 | EH domain-containing protein 1 GN=EHD1 |
| **P19320** | 0.0003 | 4.82 | 4.82 | 23 | Vascular cell adhesion protein 1 GN=VCAM1 |
| **P31689** | 0.0003 | 1.33 | 1.33 | 10 | DnaJ homolog subfamily A member 1 GN=DNAJA1 |
| **P04275** | 0.0003 | 0.7 | 1.43 | 85 | von Willebrand factor GN=VWF |
| **P16581** | 0.0003 | 7.85 | 7.85 | 14 | E-selectin GN=SELE |
| **P07996** | 0.0003 | 1.12 | 1.12 | 38 | Thrombospondin-1 GN=THBS1 |
| **P07942** | 0.0003 | 0.87 | 1.15 | 15 | Laminin subunit beta-1 GN=LAMB1 |
| **P04179** | 0.0003 | 3.29 | 3.29 | 8 | Superoxide dismutase [Mn], mitochondrial GN=SOD2 |
| **P05362** | 0.0003 | 3.71 | 3.71 | 17 | Intercellular adhesion molecule 1 GN=ICAM1 |
| **Q9P2E9** | 0.0011 | 1.1 | 1.1 | 59 | Ribosome-binding protein 1 GN=RRBP1 |
| **Q13201** | 0.0015 | 0.82 | 1.22 | 17 | Multimerin-1 GN=MMRN1 |
| **P06756** | 0.0057 | 1.14 | 1.14 | 18 | Integrin alpha-V GN=ITGAV |
| **P11940** | 0.0198 | 1.11 | 1.11 | 19 | Polyadenylate-binding protein 1 GN=PABPC1 |
| **Q9Y4L1** | 0.0317 | 1.1 | 1.1 | 43 | Hypoxia up-regulated protein 1 GN=HYOU1 |
| **Q96QV1** | 0.0318 | 0.88 | 1.13 | 11 | Hedgehog-interacting protein GN=HHIP |
| **Q9Y376** | 0.0367 | 1.16 | 1.16 | 8 | Calcium-binding protein 39 GN=CAB39 |
| **P52569** | 0.0904 | 1.76 | 1.76 | 5 | Low affinity cationic amino acid transporter 2 GN=SLC7A2 |
| **P24468** | 0.137 | 0.74 | 1.35 | 4 | COUP transcription factor 2 GN=NR2F2 |
| **Q9UNN8** | 0.2233 | 0.7 | 1.42 | 4 | Endothelial protein C receptor GN=PROCR |
| **P02794** | 0.2487 | 1.35 | 1.35 | 4 | Ferritin heavy chain GN=FTH1 |
| **Q03169** | 0.6684 | 1.42 | 1.42 | 5 | Tumor necrosis factor alpha-induced protein 2 GN=TNFAIP2 |
